# A process-based approach to cognitive behavioral therapy: A theory-based case illustration

**DOI:** 10.3389/fpsyg.2022.1002849

**Published:** 2022-10-25

**Authors:** Clarissa W. Ong, Steven C. Hayes, Stefan G. Hofmann

**Affiliations:** ^1^Department of Psychology, University of Toledo, Toledo, OH, United States; ^2^Department of Psychological and Brain Sciences, Boston University, Boston, MA, United States; ^3^Department of Psychology, University of Nevada, Reno, Reno, NV, United States; ^4^Department of Psychology, Philipps-Universität Marburg, Marburg, Germany

**Keywords:** process-based therapy, case study, processes of change, network analysis, process-based approach

## Abstract

Despite the significant contribution of cognitive-behavioral therapy to effective treatment options for specific syndromes, treatment progress has been stagnating, with response rates plateauing over the past several years. This stagnation has led clinical researchers to call for an approach that instead focuses on processes of change and the individual in their particular context. Process-based therapy (PBT) is a general approach representing a model of models, grounded in evolution science, with an emphasis on idiographic methods, network models of case conceptualization, and enhancing wellbeing. In this paper, we describe the theory underlying PBT and present a case study for how to apply PBT tools and principles to deliver process-informed and person-centered evidence-based treatment. In addition, we discuss lessons learned from our case and provide suggestions for future considerations when implementing PBT in clinical settings.

## Introduction

### Historical dominance of cognitive-behavioral therapy

For decades, cognitive-behavioral therapy (CBT) has been the gold standard of evidence-based care for many mental illnesses as defined by the *Diagnostic and Statistical Manual of Mental Disorders* (DSM), ranging from generalized anxiety disorder to eating disorders (Covin et al., 2008; Linardon et al., 2017). CBT, in turn, has built its credibility on copious data accrued from the gold standard of clinical experimental design: randomized controlled trials. The most basic design of a randomized controlled trial entails comparing the means of two groups of randomly assigned people after one group receives the active intervention and the second does not. If the treatment group mean is found to be “significantly” better than that of the control group, statistically speaking, the treatment is deemed efficacious.

Data from hundreds of randomized controlled trials have shown that protocol-based CBT leads to more symptom improvement on average compared to other interventions (e.g., Butler et al., 2006; Hofmann et al., 2012). In addition, adjunctive treatment components are constantly tested to facilitate incremental gains from CBT, such as adding motivational interviewing (Marker and Norton, 2018) or contingency management (Worden et al., 2017). Prevailing wisdom over the past few decades declared that CBT tailored to specific disorders and randomized controlled trials are the solution to mental health struggles, and most clinical research and funding accordingly have operated on this assumption (Chambless and Hollon, 1998; Tolin, 2020).

At the same time, treatment progress has been stagnating. CBT response rates have hovered around 50% for anxiety disorders for years (Loerinc et al., 2015; Springer et al., 2018), suggesting that the gold standard is not getting better, despites decades and millions of dollars of research. Furthermore, relevance of the nomothetic principles underlying randomized controlled trials to individual wellbeing is tenuous, calling into question the utility of randomized controlled trials as a means of evaluating treatment efficacy and the generalizability of study findings to individual clients (Molenaar, 2004). If the solution is not CBT protocols for disorders and randomized controlled trials, then we need to look elsewhere to ensure that clinical psychological science can adequately meet the needs of those who are suffering.

### Move toward personalized care

Over the past few decades, there has been a growing movement toward using idiographic methods (or methods that focus in the individual and their functioning rather than groups and averages) in clinical psychology research, with the ultimate objective of personalizing psychotherapy for every client (Rubel et al., 2018; Fisher et al., 2019; Levinson et al., 2021). Broadly speaking, idiographic treatment research strives to answer Paul’s famous question, “What treatment, by whom, is most effective for this individual with that specific problem, and under which set of circumstances?” (p. 111, Paul, 1967). For example, Levinson et al. (2021) identified central symptoms in individual-level networks among participants with eating disorders, then developed treatment plans by selecting corresponding modules from evidence-based treatments (e.g., emotion regulation module from dialectical behavior therapy [DBT] for feelings of shame and guilt).

Closely related to idiographic methods is a network approach to understanding psychopathology, which posits that symptoms have causal interrelationships with each other rather than are caused by a latent disease as assumed by the biomedical model (Bringmann et al., 2022). Together, these ideas reflect a conceptualization of psychopathology as networks of interrelated biopsychosocial processes and problems that form a causal and dynamic network in ways unique to each person. Thus, even if two people present to therapy with a similar complaint, their prescribed treatments may vary depending on the person’s individual network of ways of addressing problems this is causing or maintaining this complaint (Levinson et al., 2021).

### Development of process-based therapy

Against the backdrop of burgeoning interest in idiographic and network-based clinical research (Piccirillo et al., 2019), a new model of personalized evidence-based psychological treatment has emerged: process-based therapy (PBT; Hofmann and Hayes, 2019; Hayes et al., 2020a). PBT is a general approach to clinical assessment, conceptualization, and treatment, representing a *model of models*. PBT is not a new therapy. Rather, it is a new *framework* to organize evidence-based therapeutic techniques—already known to psychologists—along basic psychological dimensions relevant to human adaptation to a given context, including cognition, attention, affect, behavior, self, and motivation, as well as biophysiological and sociocultural levels (Hayes et al., 2022).

The dimensional model undergirding PBT is called the extended evolutionary meta-model or EEMM (rhymes with “dream;” Hayes et al., 2020b). Its job is to clarify the inter-relatedness among processes with respect to EEMM dimensions and levels and to facilitate finding optimal therapeutic strategies to target the most relevant processes. Analogous to a closet, the EEMM provides space to consider different aspects of one’s psychological repertoire. In much the same way that a closet is rendered useful by the clothing it contains, the utility of the EEMM ultimately depends on the existence of meaningful content but it can be considered independently of content.

**FIGURE 1:**
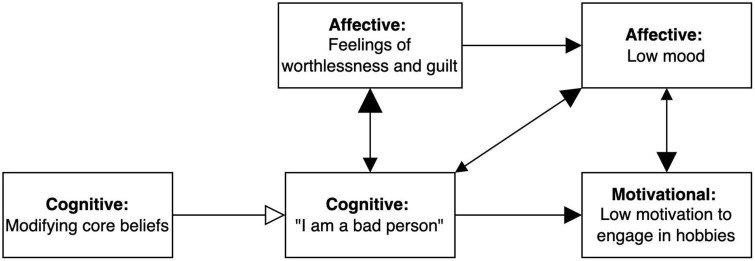
Example of network model for a specific client. Extended evolutionary meta-model (EEMM) dimensions represented by each node are bolded. Size of the arrowheads indicates hypothesized strength of the relationship (bigger arrowheads = stronger correlation), and opacity reflects direction of the relationship (opaque = positive/excitatory, transparent = negative/inhibitory).

Along with the EEMM, PBT provides tools for idiographic assessment to guide treatment planning for the individual-in-context. These tools begin with a network approach wherein clinicians identify key variables relevant to the client’s presenting problem and hypothesize about how these variables relate to one another. Using PBT graphic conventions (Hofmann et al., 2021), the direction and strength of the relationships are represented by opacity and size of arrowheads, respectively. For example, in Figure 1, the network shows that the core belief, “I am a bad person” is hypothesized to lead the client to experience feelings of worthless and guilt, low mood, and low motivation to engage in hobbies (excitatory effect depicted with opaque arrowhead), with a stronger hypothesized effect on feelings of worthless and guilt. In contrast, modifying core beliefs is thought to have an inhibitory effect on the original core belief, as illustrated with a blank arrowhead, meaning that modifying core beliefs weakens the influence of the thought, “I am a bad person,” along with corresponding downstream effects.

The intended function of PBT is to provide a theoretically coherent framework broad enough to encompass the gamut of psychotherapy orientations and furnish a lingua franca with which psychologists can use to communicate seemingly disparate ideas. Accordingly, PBT is grounded in evolution science, precisely because evolutionary principles have been postulated as a unifying theoretical framework across virtually all life science disciplines, including psychology (Mesoudi et al., 2006; Hayes and Sanford, 2015). PBT views psychopathology as maladaptation to a given context due to problems in variation, selection, and retention of biopsychosocial processes in multiple dimensions and levels (Hayes et al., 2020b). While considering the complexity and interconnectedness of problems, clinicians strive to perturbate the client’s maladaptive network of such processes while building an adaptive, self-sustaining alternative network. This is done by applying specific treatment kernels that introduce new responding (variation), identify which strategies are most adaptive for a client given their goals (selection), help clients persist in useful responding (retention), across various psychological facets (dimensions), on intrapersonal and interpersonal scales (levels), in ways that are sensitive to history, situational demands and personally relevant aspirations (context).

Although PBT emphasizes idiographic methods and network models of case conceptualization, similar to other approaches observed in clinical psychology (Fisher et al., 2019; Levinson et al., 2021), it goes beyond methodology. It also takes an explicit philosophical stance against diagnostic and symptom-driven models, instead directing efforts toward understanding clinically relevant *processes* and enhancing *wellbeing*, embodying a clear departure from randomized controlled trials and the symptom-focused tradition of clinical psychology. In other words, idiographic research focused on symptoms alone is still inadequate from a PBT perspective.

Rather, PBT entails attention to processes of change unique to the individual in their context over symptoms enumerated in a diagnostic manual (e.g., in the context of social anxiety and fear of negative judgment about physical appearance due to childhood bullying and emphasis on physical appearance in family of origin vs. fear of negative evaluation). Extending Paul’s classic question with a demand for precision, PBT instead asks, “What core biopsychosocial processes should be targeted with this client given this goal in this situation, and how can they most efficiently and effectively be changed?” (p. 2, Hayes et al., 2020a). In a way, for PBT, personalizing treatment is not an end in itself, but a means to the end of developing more effective and efficient treatments for all individuals given limited available resources.

Given its explicit philosophical and methodological stance, PBT has the potential to undermine the barriers presently facing treatment development in two ways: (Covin et al., 2008) it targets processes of change, not symptoms, and (Linardon et al., 2017) it is evaluated on the level of the individual not only the group. That is, PBT is not diagnosis-specific and hence flexible enough to be used with presentations poorly captured by DSM diagnoses (e.g., multiple co-occurring diagnoses). PBT also considers individual differences and prioritizes what works for a person in their unique context, rather than an illusory average. Moreover, the goal of PBT is to improve wellbeing not symptom reduction, the default metric against which most evidence-based psychotherapies to date have been evaluated (Linardon et al., 2017; Springer et al., 2018).

The difference between a diagnosis-based and process-based approach may be illustrated by an example. The network in Figure 1 represents a client who plausibly fits the diagnostic profile of major depressive disorder according to the DSM or ICD, given that they report such depressive symptoms as low mood, low motivation to engage in hobbies, and feelings of worthlessness and guilt. Nonetheless, assignment of a diagnosis would require a standard diagnostic interview and cannot be made based on the elements of Figure 1 alone. If, however, this client was given a diagnosis of “major depressive disorder” based on a formal assessment, choices for evidence-based treatment would include behavioral activation, cognitive behavioral therapy, and interpersonal therapy (Gloaguen et al., 1998; Cuijpers et al., 2007, 2011), and clinicians could choose among these options. However, as depicted in Figure 1, the core belief that “I am a bad person” appears to be a primary driver of the other aspects of the client’s presentation. Thus, a clinician might decide to focus on cognitive intervention strategies as a first step, predicting that it would result in downstream effects of improving other problems.

### Application of process-based therapy and current case illustration

In PBT, the treatment goal is to move clients toward adaptive growth relying on variation, selection, and retention along the EEMM dimensions and levels in a given context. In the closet analogy, this is equivalent to having the client try on different items of clothing (*variation*), identify which work for which occasions (*selection* in *context*; e.g., flip-flops for the beach and coat for winter hiking), and keep wearing appropriate clothing in specific contexts (*retention* in *context*). For the client in Figure 1, this may mean trying different cognitive strategies (e.g., restructuring from cognitive therapy, defusion from acceptance, and commitment therapy [ACT]) in different contexts (e.g., when feeling sad vs. feeling neutral) to cope with the core belief, and to be able to deploy those strategies matched to context the next time the core belief shows up.

**FIGURE 2:**
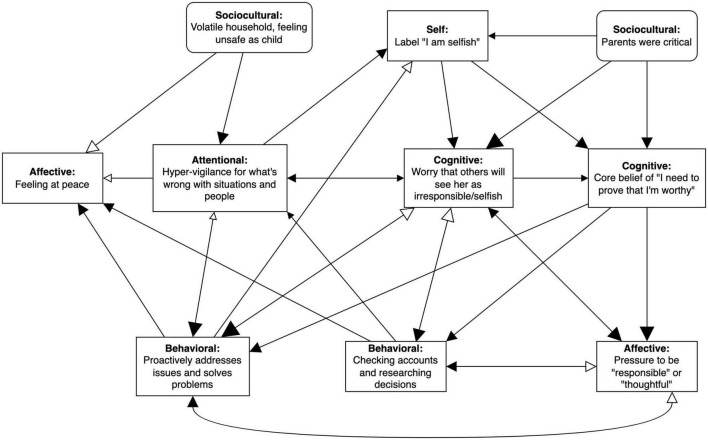
Preliminary case conceptualization for Amy. EEMM dimensions represented by each node are bolded. Size of the arrowheads indicates hypothesized strength of the relationship (bigger arrowheads = stronger correlation), and opacity reflects direction of the relationship (opaque = positive/excitatory, transparent = negative/inhibitory). Right-angled rectangles reflect manipulable variables and rounded rectangles indicate immutable moderators (e.g., historical events).

If PBT becomes the vehicle for evidence-based intervention to shift from a focus on protocols for disorders to a focus on the personalized needs of particular people, networks mapping clinically relevant processes for each person (see Figure 2 for an example) will become commonplace as a way of organizing the idiographic deployment of evidence-based treatment components or *kernels* (e.g., interpersonal effectiveness skills from DBT and interoceptive exposure from CBT). This is not an entirely new vision since it echoes the focus on functional analysis in the early days of behavior therapy, wherein general principles were applied to individual presentations, such that even if reinforcement was targeted, the form it took could be vastly different (e.g., attention vs. candy vs. money; Barlow and Hersen, 1973; Kanfer and Grimm, 1977).

There are major differences, however. The set of replicated nomothetic processes of change is now vastly larger than the behavioral learning principles (e.g., positive reinforcement in operant conditioning) that were then emphasized, which means there are many more tools and processes from which clinicians can choose on aggregate (Hayes et al., 2022). However, an idiographic lens entails precision in how processes are targeted, with the understanding that not every process is relevant to every client. For instance, distress tolerance might be important for someone with high emotional reactivity and sensitivity, whereas social skills training may be more important for someone who lacks interpersonal skills. Furthermore, unlike direct nonverbal contingencies alone, contemporary biopsychosocial processes of change are understood to be dynamic and progressive, and thus to require such analytic tools as dynamical systems analysis—wherein the state of a datapoint is assumed to be time-varying but predictable based on certain inputs (e.g., past behavior predicting future behavior)—to construct adequate functional analyses (Curtiss et al., 2021; Hofmann et al., 2021; Roefs et al., 2022), not merely classical single case designs (Hayes et al., 1999).

While the possible number of relevant processes of change is very large, evolutionary science has emerged as a parsimonious framework within which to help organize comprehensive analyses of client needs and goals across relevant dimensions and levels of biopsychosocial processes (Hayes et al., 2020a,b). Importantly, measurement tools and statistical methods are now available to help gradually turn this new approach to functional analysis into a largely empirical rather than a largely conceptual tool (e.g., Group Iterative Multiple Model Estimation [GIMME], Process-Based Assessment Tool [PBAT]; Gates and Molenaar, 2012; Ciarrochi et al., 2022; Sanford et al., 2022).

Clearly, from our vantage point, the potential of PBT is vast. We anticipate that PBT can fundamentally alter how we conceptualize mental wellbeing and design psychological treatments, leading to the development of interventions that can more effectively and efficiently meet the needs entailed in infinite human complexity. Yet, more research and clinical testing are needed to clarify and refine its application across a range of contexts. The present case illustration represents an initial step toward this effort of explicating the application of PBT principles, to provide a clinical face to the core theoretical features of the PBT research program.

## Case illustration

### Client description

To illustrate how PBT may be applied with a real-life example, we describe the course of treatment for an actual recent client, Amy, who was treated using a PBT approach to CBT. Some content details have been changed to anonymize Amy, but concepts remain functionally similar. Amy (she/her) was a cisgender White woman in her late 30 s working full-time at a university administrative job while managing a consultation business part-time at the point of study enrollment.

Based on results from the Mini International Neuropsychiatric Interview 7.0 (Sheehan et al., 1998), a semi-structured clinical interview for DSM-5 diagnoses, Amy was assigned a primary diagnosis of generalized anxiety disorder. The specific problems Amy reported were “incessant checking” of financial and email accounts, avoidance of going outside due to compulsion to report public hazards to local authorities, indecision around her own career path, and rigid adherence to standards around being “responsible.” Amy’s initial treatment goals were to clarify her values, increase physical activity, develop a plan for leaving her full-time job to focus on her consulting business, be more present in interpersonal interactions, and maintain healthy interpersonal boundaries with loved ones.

### Case conceptualization *via* network modeling

In the first two sessions of treatment, the therapist and Amy developed a preliminary network model based on her self-reported problems *via* clinical interview and discussion (see Figure 2). Each node (events represented in rectangles) or edge (relationships represented by arrows) were agreed to by Amy before being added to the network. Right-angled rectangles reflect manipulable variables and rounded rectangles indicate immutable moderators (e.g., historical events). Changeable nodes (rectangles) were defined functionally rather than topographically. For example, “proactively solving problems” covered Amy’s reporting of public hazards to authorities as well as other forms of excessive problem solving, such as making contingency plans for anticipated negative outcomes.

The selection of which nodes and edges were emphasized and retained were determined by functional analyses rooted in Amy’s primary presenting concerns: excessive checking and constant pressure to act responsibly or thoughtfully. For example, starting with excessive checking (identified behavior), the therapist and Amy explored potential antecedents and consequences contributing to or maintaining the unhelpful behavior. Together, they clarified that the pressure to act responsibly directly contributed to Amy’s excessive checking and that checking was reinforced by a sense of peace and reductions in worry about being seen as irresponsible and pressure to be responsible in the short term. Similarly, Amy hypothesized that the constant pressure she experienced to be responsible might be linked to a core belief that she needs to prove her worth and worries about being seen by others as irresponsible.

Further functional analyses were used to clarify how newly identified nodes were linked to the existing network or branched off to new areas. For instance, the self-label of “selfish” was absent at the inception of the network; it was only added after several functional analytic explorations and Socratic questioning wherein Amy realized that she had been carrying the self-label of “selfish” and the label was, in turn, driving other nodes like worry about external judgment and the core belief of needing to prove her worthiness. Typically, in the first few follow-up functional analyses, we would find that newly identified nodes linked back to existing ones. As shown in Figure 2, for example, the “selfish” self-label was hypothesized to be associated with five other nodes.

As expected, however, the further out we went from the core problem, the fewer the number of edges connected back to the network. Thus, in terms of deciding how much to expand the network, we used the recommendation outlined in the PBT guide, *Learning PBT*: “as complex as necessary and as simple as possible” (p. 20, Rubel et al., 2018). In other words, we considered relevance to the network and presenting concern to give us a sufficiently complex understanding of Amy’s struggles to inform treatment planning, while letting go of other variables that may have been related to Amy’s struggles but did not incrementally contribute to treatment planning.

As an example, an early iteration of Amy’s network included a history of learning difficulties that she believed contributed to her attentional bias toward things going wrong, but through discussion, it seemed that the more pertinent contributor was her upbringing in a volatile household. Moreover, learning difficulties did not directly relate to other parts of her network or shape treatment planning beyond that explained by existing nodes (e.g., critical parents), thus, it was excluded from the first draft of Amy’s network.

**FIGURE 3:**
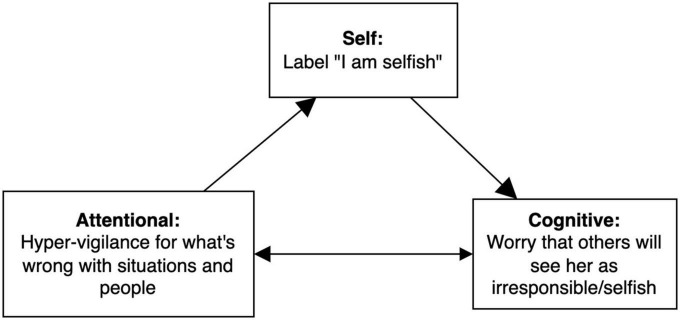
Self-amplifying loop from Amy’s broader case conceptualization. EEMM dimensions represented by each node are bolded. Size of the arrowheads indicates hypothesized strength of the relationship (bigger arrowheads = stronger correlation).

Once the network was completed, self-amplifying subnetworks were identified to clarify potential treatment targets. As can be seen in Figure 2, Amy’s preliminary network contains several self-amplifying loops or subnetworks that are self-maintained, one of which is illustrated in Figure 3. In this self-amplifying loop, occurrence of the self-concept of “selfish” leads Amy to worry that others will perceive her as irresponsible or selfish, which then leads her to focus on problems in the physical or social environment in a hyper-vigilant way. This attentional bias, in turn, leads her to be more likely to view herself as “selfish” and to worry even more about external judgment. Because this part of the network is self-amplifying, no external input is needed to maintain the self-criticism, worry, and attentional bias cycle, making it especially critical to disrupt it during intervention.

**FIGURE 4:**
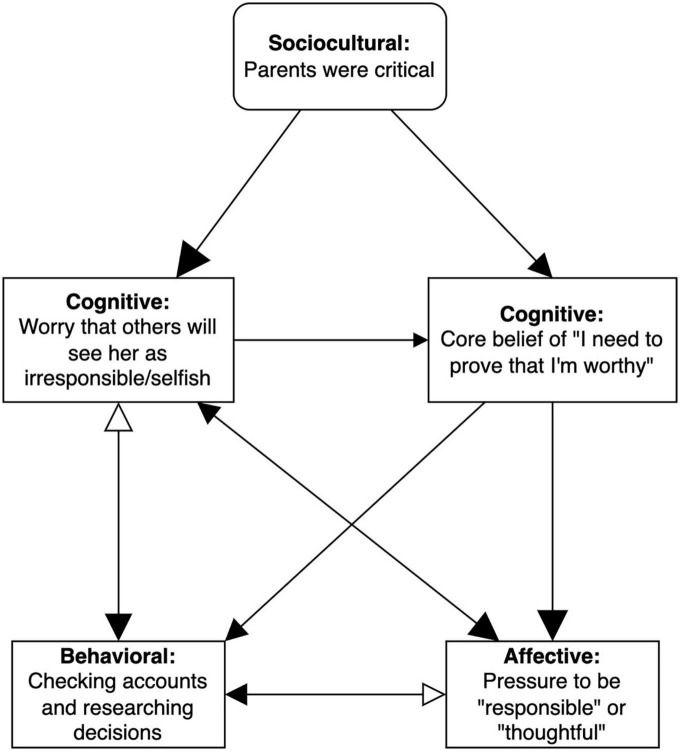
Subnetwork with inhibitory effects. EEMM dimensions represented by each node are bolded. Size of the arrowheads indicates hypothesized strength of the relationship (bigger arrowheads = stronger correlation), and opacity reflects direction of the relationship (opaque = positive/excitatory, transparent = negative/inhibitory). Right-angled rectangles reflect manipulable variables and rounded rectangles indicate immutable moderators (e.g., historical events).

A different example of a subnetwork is shown in Figure 4. In this case, inhibitory arrows contribute to the self-perpetuating pattern. A moderator from Amy’s past (“parents were critical”) continues to drive the worry of being viewed as selfish and the core belief that she needs to prove herself worthy, despite the inhibitory influence of checking behavior. For instance, worry that others will see her as selfish brings up fear of making an “irresponsible” decision, which leads Amy to repeatedly check online accounts and to research decisions. These compulsive behaviors are negatively reinforced in the short term because they decrease her worry of being viewed as selfish and fear of making a poor decision. Without the sociocultural moderator of critical family members, this should dampen the self-perpetuating cycle, but Amy’s history keeps her worry and core belief active (note the relatively bigger arrowheads reflecting stronger hypothesized influence), such that the loop persists despite short-term reduction in worry and fear. Furthermore, the subnetwork functions on a short timescale (daily), such that a similar subnetwork with a monthly timescale may actually show that checking *increases* worry.

Of note, a key node in Amy’s network is worry about being perceived as irresponsible or selfish, which coheres with the primary assigned diagnosis of GAD, potentially raising questions about the incremental utility of a network case conceptualization. Although a PBT approach may ultimately identify the same broad treatment target as a DSM diagnosis, which is “worry” in this case, the distinct feature of PBT is that it also identifies the downstream and upstream variables tied to worry (e.g., attentional bias for things going wrong, “selfish” label, pressure to be responsible, excessive checking, problem solving; see Figure 2) that may elude a GAD diagnosis alone. The practical implication of this is that the PBT approach would provide more personalized treatment targets for Amy (e.g., acceptance of perceived pressure to be responsible and mindfulness training to increase attentional flexibility) rather than recommend general evidence-based approaches for worry like worry time or cognitive restructuring.

### Assessment of treatment progress

The network is a dynamic, transitory system to guide case conceptualization and treatment progress (Fried et al., 2017; Curtiss et al., 2021; Roefs et al., 2022). Its purpose is to capture the complexity of the client’s problems and to serve as a way to identify causal influences, identify treatment targets, and monitor treatment progress. In a PBT approach, the network is viewed as constantly changing and thus needs to be re-examined on a regular basis, especially during therapy.

In order to characterize the network of processes of change empirically, over the course of treatment, Amy completed personalized ecological momentary assessment (EMA) items four times a day based on her case conceptualization (see Table 1). EMA items were rated using a visual analog scale from 1 to 100. The wording and frequency of items are presented in Table 1.

**TABLE 1:** Personalized ecological momentary assessment items over the course of treatment.

	Start	End	Frequency	Items
Behavioral goals	Baseline	1-month follow-up	Once a day	• Since the start of today… How many minutes did you use your Mail app for? • Since your last response… How many minutes have you been engaged in physical activity? Estimate as best as you can.
Network nodes (Initial)	Baseline	Midtreatment	4 times/day	In the past 3 h, to what degree did you… • Focus on what’s wrong (with yourself, others, situations, environments, etc.)? • Feel at peace? • Feel pressure to be responsible or thoughtful? • Act on a compulsion to solve problems or take care of others?
Network nodes (Revised)	Mid-treatment	Posttreatment	4 times/day	In the past 3 h, to what degree… • Did you feel empowered? • Did you act in ways that serve your wellbeing? • Did you demonstrate flexibility regarding standards? • Were you aware of how you were feeling? • Did you build connection with people?
Progress	Mid-treatment	Posttreatment	Once a day	Considering your choices and actions in the past 3 days, to what extent… • Are you making progress on your goals? • Do you trust yourself?

#### Daily self-report items

Assessment initially included behavioral goals (duration) and key nodes in Amy’s network determined in the first two sessions of treatment. As Amy began to practice new skills and add adaptive nodes to her existing network, we replaced the original prompts with items describing her new adaptive network and assessing progress vis-à-vis her goals. All items were personalized to Amy’s presentation and developed collaboratively with her input. That is, items were only added to the daily assessment if Amy agreed that they would be relevant to her wellbeing. Table 1 lists these daily self-report items from baseline to the end of treatment.

#### Network analysis

Amy’s network data were analyzed using the Group Iterative Multiple Model Estimation (GIMME; Gates and Molenaar, 2012) package in R version 4.2.0 (R Core Team, 2022). GIMME is an idiographic algorithm applied in a structural equation modeling (SEM) and Vector Autoregressive (VAR) framework that identifies how variables of interest relate to each other and evaluates the strength of the relationship among variables. GIMME accounts for longitudinal data and corresponding autoregressive effects by estimating the unified SEM (Lane et al., 2021), which permits evaluation of contemporaneous and temporally lagged relations among variables of interest simultaneously. In addition, contemporaneous directionality is indicated when X at time *t* explains more variance in Y at time *t* than Y at time *t* does in X at time *t*, after addressing other variables in the model, including autoregressive effects. Contemporaneous directionality is not equivalent to causality given lack of experimental control, but it can be generally predictive of temporal relationships in smaller temporal windows than those used to collect the EMA data.

In GIMME, individual-level models are first estimated independent of any group-level data, and group-level (and, if relevant, subgroup level) models are subsequently generated based on individual-level models, retaining edges only if they apply to the majority of individuals in the sample. In the present case, because there was only one participant, only an individual-level model was generated, and the group-level step produced the exact same results as the individual-level model given an N of 1. The subsequent model fitting for the individual-level model is resolved when there is an excellent model fit based on two of four model fit indices: root mean square error of approximation (RMSEA) < 0.05, standardized root mean square residual (SRMR) < 0.05, non-normed fit index (NNFI) > 0.96, and comparative fit index (CFI) > 0.95 (Bentler and Bonett, 1980; Bentler, 1990; Steiger, 1990; Hu and Bentler, 1999; Brown, 2015). Model estimation and missing data were handled using full information maximum likelihood. More details on GIMME procedures can be found in Gates and Molenaar (2012).

#### Treatment plan

In the third session, the therapist reviewed the conceptual network model with Amy (Figure 2), confirming with her that the model was accurate to her experience. In the third session, the therapist reviewed the conceptual network model with Amy (Figure 2), confirming with her that the model fit with her experience and with her own conceptualization of her struggles. This is in the spirit of the idiographic PBT approach to assessment as opposed to a top-down “expert clinician” approach. Based on this understanding of Amy’s struggles, the therapist and Amy collaboratively developed a treatment plan. Based on Amy’s goals and network, they agreed that treatment would start with targeting inflexibility around personal standards (e.g., needing to be responsible or worthy) and attentional control (e.g., being more present).

Amy’s rigidity around personal expectations manifested in significant time spent on problem solving, checking online accounts, and researching prior to decision making in response to worry and her core belief. For example, her need to be responsible or perceived as responsible led to her spending hours comparing household products before purchasing one. Thus, we hypothesized that if Amy was able to hold personal standards more lightly, she would respond to them in more values-consistent and wellness-enhancing ways.

Amy also had difficulty regulating her attention, primarily focusing on negative aspects of situations and people—partly due to her chaotic childhood in which this hypervigilance was adaptive. As an adult, however, the hypervigilance reinforced the self-concept that she was “selfish,” led to worry about negative evaluation, and motivated excessive preemptive problem solving. We hypothesized that if Amy learned to shift attention intentionally, she would still retain the ability to be vigilant, when necessary, but also be present and open to other sources of data about herself and those around her (e.g., she is kind, others find her charming) when not exclusively attuned to negative concepts.

### Treatment description

At the end of the first three sessions of collaborative case conceptualization and treatment planning, the therapist provided psychoeducation on how standards govern behavior when they are held rigidly and asked Amy to think of examples where standards may be driving her behavior (functional analysis from CBT; e.g., Barlow et al., 2017). Amy noted that the expectation that she “needs to respond to people as quickly as possible” was a motivator of her incessant checking behavior. The therapist then assigned homework to Amy to identify 5-10 standards she follows and ways in which those standards influence her behaviors to increase self-awareness through self-monitoring.

In session 4, Amy reported that she had discovered many standards that were influencing her behavior and, with this awareness, was able to respond to them more flexibly using cognitive defusion from ACT. For instance, Amy had gone on a vacation in between sessions and noticed the standard, “I need to make the most of my vacation,” which would have typically led her to pack her schedule with back-to-back activities. Instead, once she noticed this standard, she intentionally chose to enjoy a slow breakfast in the morning and only started exploring the city in the late morning, demonstrating healthy *variation* in responding (i.e., potentially useful responses outside her existing repertoire).

To facilitate *selection* of adaptive responding, the therapist asked Amy to track the consequences of this new behavior. For instance, Amy noted that she enjoyed her day more and relaxing her standards even gave her the opportunity to try out unplanned activities, which satisfied her desire for adventure. Directing Amy’s attention to these outcomes was important for helping Amy determine if her new responses were adaptive (and thus should be selected for retention in her repertoire) or maladaptive (and thus continued variation was needed). In other words, variation alone is inadequate. Amy also needed to evaluate the utility of any new responding to eventually shape a more salubrious set of responses.

In addition, Amy said that tracking her behaviors had been helpful for supporting desired behavior change. To capitalize on momentum toward positive behavior change and to reinforce flexible responding, the therapist asked Amy to practice doing behaviors that served her wellbeing for homework. Note that by defining the behavioral task functionally (i.e., “serve wellbeing”), the therapist was giving Amy room to continue *varying* forms of enhancing wellbeing (e.g., waking up late, going to the gym, and connecting with old friends) and to ultimately *select* those that were most effective in meeting her needs.

**FIGURE 5:**
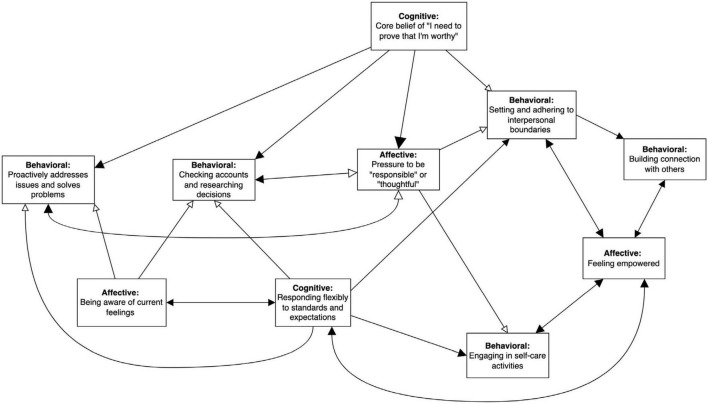
Subnetwork with adaptive nodes added after start of treatment. EEMM dimensions represented by each node are bolded. Size of the arrowheads indicates hypothesized strength of the relationship (bigger arrowheads = stronger correlation), and opacity reflects direction of the relationship (opaque = positive/excitatory, transparent = negative/inhibitory). Right-angled rectangles reflect manipulable variables and rounded rectangles indicate immutable moderators (e.g., historical events). Reinforcing valence of “feeling empowered” is indicated by double-headed excitatory arrows with “responding flexibly to standards and expectations,” “setting and adhering to interpersonal boundaries,” “building connection with others,” and “engaging in self-care activities.”

In the next three sessions (Worden et al., 2017; Marker and Norton, 2018; Tolin, 2020), Amy reported that identifying and responding flexibly to standards had been “empowering” for her. She provided examples of explicitly communicating her needs, asking for help from others, driving instead of walking when it was cold, saying no to burdensome requests, and delaying responding to emails. Amy noted that these behaviors were consistent with her values (she had previously done values clarification work through a leadership training) and was able to generalize flexible responding to rules to various life domains. Moreover, Amy observed that the feeling of empowerment she derived from flexible responding was “self-reinforcing.” The reinforcing function of the feeling of empowerment, along with other new behaviors, was added to Amy’s network conceptualization (see Figure 5). The eventual objective was to transition from Amy’s stable maladaptive network to a sustainable adaptive network.

During this time, Amy made two significant life decisions. The first was to resign from her full-time administrative job to focus on her consulting business and the second was to undergo an elective surgery to improve her physical health, which generated anticipatory excitement and indecisiveness, eliciting the familiar pressure to act responsibly or thoughtfully. These decisions resulted in re-activation of her original network (e.g., increasing worry that others will judge her decisions negatively), though Amy clarified that the decisions was consistent with her values and could conceptualize them as forms of self-care, engaging with her newer “adaptive” subnetwork (see Figure 5).

At the end of session 7, the therapist and Amy reviewed personalized items to track *via* EMA, which led to a revision of the EMA survey (see Table 1). The revisions clarified Amy’s current treatment goals and added progress items to monitor progress toward her updated goals. The reason for the EMA review was that Amy had already achieved her early treatment goals of clarifying her values, increasing physical activity, leaving her full-time job, being more present in interpersonal interactions, and maintaining healthy interpersonal boundaries with loved ones.

Starting in session 8, treatment became more focused on *retaining* newly selected behaviors and further enhancing wellbeing, after Amy indicated that she would like to continue treatment to work on practicing healthy detachment from thoughts and feelings, structuring her life in a more balanced way (e.g., having hobbies outside of work), and being more intentional with her actions. At this time, Amy reported continued reduction in problematic behavior (e.g., reporting public hazards once a week vs. multiple times a week) and an increase in helpful behaviors, such as building interpersonal connections, being more present, and practicing detachment from her expectations and emotions—which, in turn, facilitated valued action.

At the same time, Amy experienced novel stressors related to her significant decisions: managing a business on which she was now primarily financially dependent and decreased access to values-based activities (e.g., socializing with friends, attending public events, and exercise) due to recovery from surgery. For example, she worried about finding health insurance, filing taxes as a business owner, and maintaining financial stability. Thus, although Amy had retained selected skills, her context shifted, providing a useful test for the resilience of Amy’s adaptive network in Figure 5: would she revert to maladaptive responses (e.g., compulsive checking) or be able to engage in new strategies she had been practicing?

Amy reported improved ability to handle some of these new stressors due to increased “trust in [herself]” to make healthy decisions and evaluating the effectiveness of those decisions with respect to her values. However, she also observed that, in other instances, she was “still trying to prove herself by overcommitting,” which was related to her core belief that she needed to prove her self-worth to others. Treatment was thus spent on reinforcing referencing values rather than standards when making choices in the presence of distress and reflecting on how well she was able to accomplish this since the last session.

In session 12, Amy brought up the issue of struggling to keep up with her values, and it became apparent that Amy had been trying to maximize her values to the extent that doing so felt overwhelming. In addition, she was so concerned with planning her “best life” that she was having difficulty being present when engaging in valued activities. These problems, while different in form, were functionally similar to Amy’s original struggles of attentional rigidity and compulsive problem solving, indicating that Amy had indeed reverted back to parts of her old network. While Amy reported that she was able to respond flexibly to standards and preset boundaries, she found attentional flexibility more challenging. Accordingly, the therapist and Amy reviewed attentional control and mindfulness skills and being discerning about which values to enact.

The final three sessions 16–18 consisted of reflecting on helpful strategies, the contexts in which they worked, ways to generalize and evaluate effectiveness of strategies, progress made, and areas to continue to strengthen. In particular, the sessions focused on the sustainability of changes she was implementing.

### Treatment outcomes

#### Daily self-report items

**FIGURE 6:**
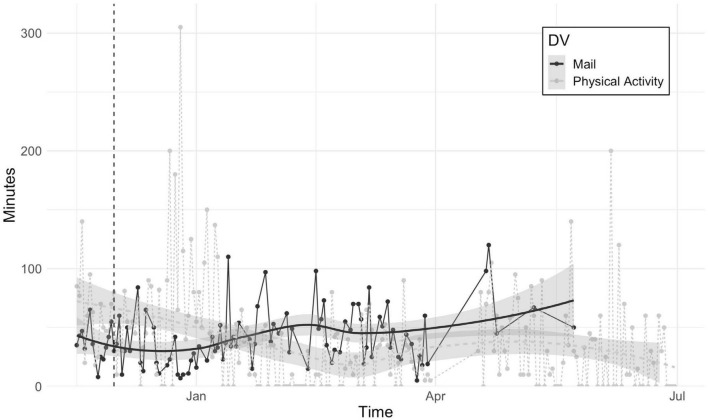
Minutes spent using mail app and engaging in physical activity over the course of treatment. The dashed vertical line indicates the start of treatment. Shaded area shows 95% confidence intervals for best-fitting lines.

Ecological momentary assessment items included Amy’s initial behavioral goals of decreasing use of the email app on her phone and increasing physical activities and, in the latter part of treatment, progress toward new goals. Figure 6 shows changes over time in Amy’s initial behavioral goals, which appears to show greater variability in physical activity over time (with less activity in March due to recovery from surgery) and a steady increase in use of her email app. These behavioral outcomes along would suggest little response to treatment, though it is possible their function changed over time given Amy’s significant contextual shift (e.g., resigning from job to run coaching business full-time). For instance, Amy reported that checking emails became more about managing the transition from part-time to full-time consulting rather than to alleviate worry.

**FIGURE 7:**
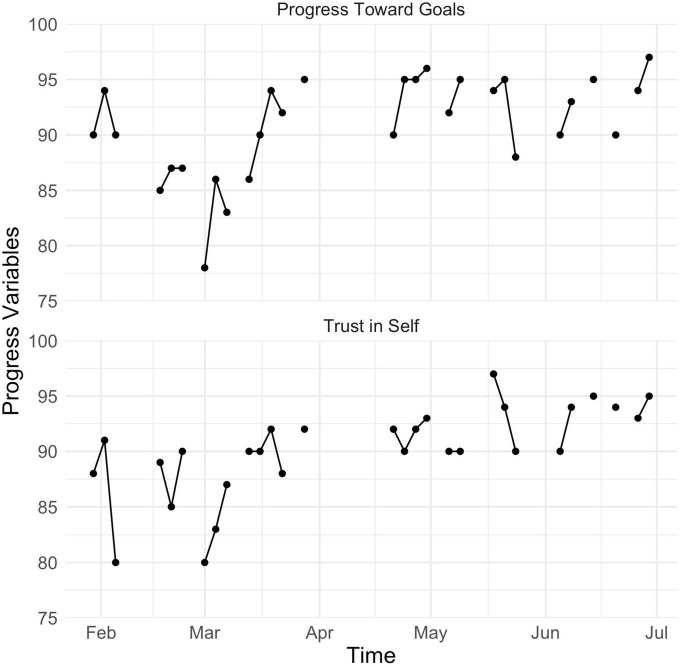
Amy’s scores for self-rated progress toward goals and degree of trust in herself from mid- to posttreatment.

We also tracked Amy’s degree of progress toward goals and trust in herself (e.g., to make healthy decisions and engage in valued action) daily (see Figure 7). Even though Amy rated herself highly on progress toward goals and trust in self (scores were around 90 out of 100) at the beginning of assessment, there was more fluctuation in the first 2 months (February to April) relative to the latter 2 months of tracking (May to July), suggesting that these indices of progress became more consistent over time.

#### Group iterative multiple model estimation networks

We used the GIMME algorithm to empirically map parts of Amy’s network during the start and end of treatment (97 observations over 36 days and 233 observations over 169 days, respectively; note that data were collapsed over weeks and do not represent any specific timepoint). Due to participant burden of responding to multiple items multiple times a day, we modeled approximately five nodes of the network for each period. To evaluate consistency between our hypothesized network and the data-driven network, we inspected presence, direction, and strength of the relationships between nodes, noting any significant discrepancies (e.g., direction of arrow was opposite to prediction). The plan was to clarify these discrepancies with the client and adjust the treatment plan accordingly, before continuing empirical testing to see if our revised hypotheses led to more adaptive responding.

**FIGURE 8:**
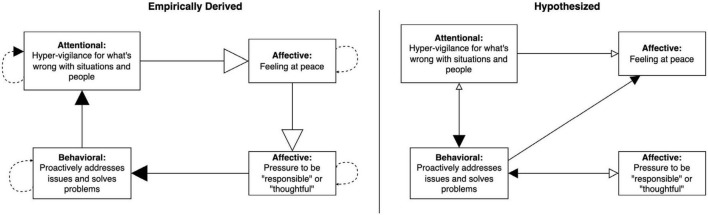
Group Iterative Multiple Model Estimation (GIMME)-derived network showing contemporaneous and lagged relationships among EMA items completed by Amy in the first month of treatment on the left, compared to our hypothesized network on the right. Solid lines, contemporaneous; dashed lines, lagged; solid arrowhead, excitatory or positive relationship; blank arrowhead, inhibitory or negative relationship. Size of arrowheads corresponds to strength of relationship estimated by GIMME analyses. Beta estimates are presented in Supplementary Table S1 in Supplementary material.

The network on the left of Figure 8 shows that, at the start of treatment, Amy’s hyper-vigilance for things going wrong was associated with feeling less peaceful and more attentional bias at the next measurement occasion. In turn, feeling at peace was related to less perceived pressure to act responsibly or thoughtfully. In other words, the hyper-vigilance had a suppressive influence on what could have been a buffer for feeling pressure to be responsible, which was itself linked to more problem solving. Problem solving was associated with greater attentional bias toward problems in her environment, completing a cyclical pattern of looking out for problems and feeling an obligation to immediately resolve them.

Our hypothesized network on the right of Figure 8 indicates several discrepancies compared to the empirically formulated network. For example, we had missed the suppressive influence of feeling at peace on the pressure to be responsible and misidentified the direction of the relationship between hypervigilance and problem solving. In terms of treatment planning, this means that we could have done more to practice strategies to bolster Amy’s feelings of peacefulness or to address the pressure to be responsible. In this specific instance, the intervention plan of focusing on attentional regulation and flexibility with respect to standards ended up targeting overlapping pathways (e.g., attentional regulation may have helped Amy to feel more at peace, which led to less problem solving through less pressure to be responsible), which may explain why treatment was still effective. However, our hypothesized processes of change were inaccurate and understanding how change actually occurred has implications for which strategies would be most helpful for targeting potential resurgence of maladaptive behaviors in the future.

**FIGURE 9:**
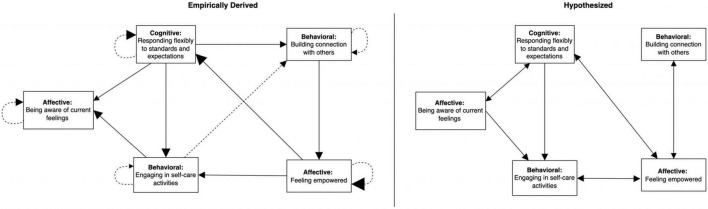
Group Iterative Multiple Model Estimation-derived network showing contemporaneous and lagged relationships among EMA items completed by Amy in the final few weeks of treatment on the left, compared to our hypothesized network on the right. Solid lines, contemporaneous; dashed lines, lagged; solid arrowhead, excitatory or positive relationship. Size of arrowheads corresponds to strength of relationship estimated by GIMME analyses. Beta estimates are presented in Supplementary Table S2 in Supplementary material.

As the original nodes became less relevant to Amy’s treatment given that she was building new skills, we began tracking new processes in the latter part of treatment to assess whether Amy was able to maintain a new adaptive network in the presence of stressors. Using GIMME, we found that responding flexibly to standards and expectations was associated with more awareness of current feelings, self-care, and connection building, demonstrating validity that cognitive flexibility was an important skill for Amy (see Figure 9). Furthermore, it resulted in more flexible responding at the next measurement occasion, suggested it was self-sustaining, similar to awareness of feelings. The lagged self-recursive relations suggest that the more Amy practiced these skills, the more she was able to access them at subsequent occasions.

Building connection and being present with others not only led to more connection building at the next timepoint but also was associated with an increase in feeling empowered. Feeling empowered also resulted in more of the same at the next assessment, corroborating Amy’s self-report that empowerment was self-reinforcing. Feeling empowered was itself linked to more cognitive flexibility and self-care. By looping back to cognitive flexibility, a three-node self-amplifying subnetwork resulted (cognitive flexibility → building connection → empowerment).

A second self-amplifying networks was identified empirically by GIMME through the feeling of empowerment node because self-care was related to more awareness of current feelings and, at the next timepoint, building connection with others and then back to empowerment.

All the relationships in these networks were excitatory and every node was endogenous (had an arrow feeding into it). Taken as a whole, the entire network and these two self-amplifying subnetworks in particular, seem likely to be stable and self-perpetuating. Clinically, we observed that Amy continued to access skills of cognitive defusion and building connection in the presence of significant stressors, which supports that possibility.

In comparison to the empirical network, the hypothesized network missed that cognitive flexibility and self-care might be positively linked to building connection with others and that self-care was more likely to drive awareness of current feelings that the other way around (see Figure 9). While this discrepancy would not significantly change our treatment plan, we might place less emphasis on practicing mindfulness of feelings as its own end if Amy did not find this to be a helpful skill. In this case, Amy reported that it was helpful in itself, even though it did not influence other nodes as we had hypothesized.

## Lessons learned

### Burden of repeated tracking on client

Although Amy was initially compliant with completing EMA surveys four times daily, she reported that she found it stressful and difficult to keep up with the surveys on several occasions, resulting in more missing observations toward the end of treatment. This was the primary reason we chose to minimize the number of items administered in each survey. Ideally, we would track every node in Amy’s networks, but this was not pragmatically feasible.

The potential burden of tracking for some clients warrants judicious planning, to the extent possible when working with complex dynamic systems, of variables that can be consistently assessed over the course of treatment. This set of variables should include problematic and desired behaviors and progress toward established treatment goals to capture a comprehensive picture of client functioning and wellbeing. Furthermore, clinicians should generally strive eliminate redundancy and select maximally orthogonal items to explain as much variance with as few items as possible. This may require some form of pilot testing in the first couple weeks of treatment to empirically determine which items to retain.

However, as a caveat, by definition, a dynamic system changes over time, and our preliminary best guesses are necessarily based on a snapshot of the client at intake. Thus, even with careful planning, clinicians may still need to adjust assessment based on client’s evolving needs and goals to meet the constant objective of improving their wellbeing and reducing suffering. Furthermore, given empirical considerations, clinicians also need to be aware of the potential ramifications of administering fewer items, especially if classical psychometric theory is a primary guide. Psychometric validity may be compromised when as few as one item is used to capture a multifaceted psychological construct, increasing measurement error as traditionally viewed within psychometrics (Piccirillo et al., 2019; Bringmann et al., 2022). Possible ways to circumvent these issues include incorporating passively collected data (e.g., from wearables and smartphones), reducing number of assessment timepoints, and using planned missing data designs and imputation methods for multivariate time series data (Piccirillo et al., 2019; Bringmann et al., 2022)—though implementation of these strategies must be theoretically and methodologically defensible, especially since the idiographic basis of PBT challenges features of traditional psychometrics (Ciarrochi et al., 2022).

### Duration of assessment

Group Iterative Multiple Model Estimation requires a minimum of 60 datapoints (ideally 100) to reliably estimate a network for a given individual (Lane et al., 2019). Considering our previous point about participant burden, one might recognize a tension between getting data quickly to empirically verify hypotheses as soon as possible and minimizing the number of times a client has to complete a daily EMA survey. For instance, we could collect the necessary data for GIMME analyses in approximately 2 weeks with four daily assessments or take a month with two daily assessments. In addition, aside from addressing pragmatic concerns, using varying timescales impacts the construct validity of variables being measured as viewed in a psychometric context (Bringmann et al., 2022). For example, assessing rapidly shifting constructs less frequently (or vice versa) increases measurement error as traditionally conceived. Thus, clinicians must bear in mind the hypothesized rate of change of variables of interest when determining the delivery schedule of EMA items. While Bayesian methods may eventually permit fewer observations for analysis due to initial consideration of clinically driven starting estimates (as opposed to starting from zero information; Burger et al., 2021), most currently available statistical methods still require significant client input *via* intensive longitudinal assessment.

### Changing relevance and function of personalized items

Amy was very involved in the case conceptualization and assessment process, providing input into which items she hypothesized would be helpful to track and were most relevant to her goals. As Amy expanded her network over the course of treatment—adding adaptive nodes and decreasing engagement in maladaptive nodes—the most relevant items changed accordingly. For example, Amy initially tracked how much she felt pressure to be responsible or thoughtful, which contributed to compulsive problem solving and checking, but later was more interested in tracking the extent to which she acted in ways that served her wellbeing as her compulsive behaviors decreased over time and self-care activities gradually increased.

The decision to drop items with low frequency was clinical and pragmatic. First, the therapist and Amy decided that it would be more helpful to focus on behaviors that Amy wanted to retain over time from a strengths-based perspective. Second, Amy found completing surveys four times daily for more than five to eight items burdensome, so we needed to distill the EMA items down to the most relevant and essential variables. However, the change in variables measured presented a research problem: how do we measure progress using different metrics at pre- and posttreatment? Our solution to this quandary was to introduce progress items (see bottom row of Table 1) that were designed to capture overall progress with respect to Amy’s overarching treatment goals. These goals were important in that the closer Amy was to accomplishing these goals, the more she was satisfied with the direction of her life.

### Failure of topographical behavioral variables to capture adaptive change

Amy initially presented as extremely high-functioning and had already been reporting high frequency of desired behavior (i.e., exercise), which made seeing a further increase in physical activity improbable. In addition, the function of her use of the email app on her smartphone changed over the course of treatment, such that its consistent frequency did not reflect a constant state. Specifically, Amy quit her full-time job during treatment and dedicated more time to building her consulting business. Thus, the initial function of checking emails to reduce anxiety about being perceived as irresponsible or selfish shifted to approaching her value of being financially stable and growing her business. These interpretations are supported by Amy’s self-report that she was no longer immediately responding to emails and more willing to wait until it was a convenient time to do so. In this case, even though the variable remained the same, its meaning and relevance to Amy’s wellbeing had changed. Said in another way, this case revealed once again that topographically defined behavioral goals are not necessarily the same as functionally defined outcomes. The topography-function discrepancy in assessment is one reason to focus on processes of change. In this case the therapist and Amy also introduced variables that were a shorthand for positive change Amy had made in treatment.

### The importance of empirical case networks

Knowing *that* treatment works is not the same as knowing *how* treatment works. It would have been reasonable to assume because our clients improved in expected ways, that our treatment plan accurately represented core struggles and processes of change. GIMME, used as an empirical case conceptualization tool, showed otherwise. In the present case, this discrepancy turned out to be largely inconsequential—given that our treatment plan targeted pathways that overlapped with those indicated by the empirically derived network—but that should not be assumed. Understanding how treatment works for specific individuals is important once a process-focus is adopted and, thus, the larger lesson of the present case is that conceptual network analysis should not be relied on as the sole evidence of how processes of change apply to a case. Empirical methods need to be developed and used in conjunction with clinical judgment (Burger et al., 2021).

With GIMME, we were able to clarify the processes of change involved in Amy’s response to treatment by checking parts of our hypothesized networks against empirically derived ones. Generally, while we found that had accurately hypothesized certain relationships, we sometimes overlooked relations or misidentified their directionality. The oversight, in Amy’s case, did not warrant an overhaul of our treatment plan, but it is entirely plausible that it could have. For example, if flexible responding to standards exerted no influence on any other node, then we would have needed to examine if Amy was properly practicing flexible responding to standards or if another cognitive strategy would have been more effective. At the same time, most analysts currently will hold empirically derived networks accountable to such traditional psychometric issues as measurement error, so clinicians will need to optimize their data collection setup for hypothesis testing beforehand. Moreover, once analyses have been completed, clinicians should verify empirical findings against clinical observations and client self-report to ensure they maintain a balanced and useful case conceptualization.

The inconsistency between conceptual and empirical networks points to a long-standing weakness of functional analysis, underscoring the need to take into account multiple sources of information (Steiger, 1990). Functional analysis is still advocated in clinical psychology (Haynes et al., 2011), but it is not commonplace because it has remained more of an art than a science. In applied behavior analysis, functional analysis grew substantially when it integrated empirical methods by using an alternating treatment design (Barlow and Hayes, 1979) to identify idiosyncratic reinforcers for undesirable behavior (Iwata et al., 1982). Unfortunately, if clients are even minimally verbal, that direct contingency approach plumets in its ability to reliably identify the functions of actions (Belisle et al., 2017). Another functional analytic approach needs to be found that can accommodate the degree to which verbal/cognitive processes operate on and alter other processes. The present study suggests that the use of EMA data on processes of change analyzed empirically as an idiographic complex network may be that pathway forward. If broadly used, such an approach might provide data person by person on the use of precise clinical interventions linked to processes of change; in effect, building a constellation of cases that can help to inform future case conceptualization.

There will be multiple problems to overcome before that future is fully available, however. For example, when developing and interpreting client networks, clinicians need to clarify the timescale of the relationships among nodes. Hypothesized networks can readily specify variable temporal lags (e.g., healthy eating — > feeling energetic could take a week, whereas feeling energetic — > engaging in hobbies may occur over minutes) but existing network tools such as GIMME often assume that data are measured at equal intervals. Self-report items are difficult to assess on a granular level, but physiological data from wearables (e.g., heartrate variability) may be measured many times each second. In the present case, the empirically derived GIMME networks appeared to be less sensitive to temporally proximal relationships, such as the possible negative reinforcement of compulsive checking through reduction of anxiety. Thus, the generation of adequate idiographic biopsychosocial complex networks are far from turnkey at the present time.

## Summary and recommendations

In this report, we provided a case illustration for using a process-based approach to case conceptualization, treatment planning, and treatment delivery to a client, Amy, in an outpatient setting. Generally, results from assessment data indicated that, despite initial high levels of functioning, Amy accumulated new skills targeting key nodes in her initial network that appeared to be resilient against external stressors and further improved her functioning. The specific steps of this case included:

(a) using a network comprised of interrelated variables rather than diagnostic labels and topographical symptoms to describe our client’s presenting problem;
(b) collaborating with the client on her case conceptualization, using her wording and input as much as possible;
(c) administering EMA items on a daily basis to collect intensive longitudinal data to evaluate treatment progress;
(d) designing treatment plan to target nodes that appeared to be contributing to other struggles;
(e) using idiographic statistical analysis to verify hypothesized networks;
(f) adjusting the treatment plan in response to empirical data and contextual shifts (should also be done in response to lack of progress, which did not occur in this case); and
(g) aiming to establish adaptive network and assessing sustainability and resilience of new network.

In some ways, because of the rigor, consideration, and expertise that went into our application of PBT, this case illustration may be considered a current “best-effort” example of how to implement PBT. We recognize that many clinicians may not have the time or bandwidth to monitor daily EMA data from clients, learn advanced statistical techniques, and generate multiple networks for each of the many clients on their caseload. Furthermore, we ourselves observed several aspects that we would do differently in the future, as noted in the *Lessons Learned* section above. Yet, our objective in providing this case illustration along with the lessons learned along the way was precisely to show that delivering PBT is an iterative process; no clinician will ever consistently deliver PBT “perfectly” given the complexity of our clients and fallibility of human clinicians.

Nonetheless, we believe that implementing principles and core pieces of a process-based approach is feasible. First, clinicians can create networks with their clients to better understand how their problems relate to and drive each other. This could supplement or replace the standard intake interview that most clinicians already do. Second, clinicians can design treatment plans based on the network, selecting among techniques they already have in their therapeutic arsenal. The difference is that the application of these techniques would be process-based, individually tailored, and hypothesis-driven—thereby more precise—rather than diagnosis-focused, similar to making a specific recommendation to eat more leafy greens over asking someone to eat more fruits and vegetables. Thirdly, clinicians who use routine outcome monitoring can use those existing items to test their hypotheses to the extent that the items are relevant and modify their approach accordingly. These changes would not require immense commitments and bring clinicians closer to a process-based approach. Finally, there is no reason that EMA and statistical tools cannot be automated in the form of apps, software, and clinical tools, making the applied task far easier.

Ultimately, through iterative learning, curiosity, cumulative skill acquisition, and the development of technical supports, clinicians will become better able to implement process-based principles with facility and build on existing methods to improve their delivery of idiographic, empirically grounded, and process-based care. It will gradually become easier to engage in intensive longitudinal data collection by automating passive collection of data, administration of self-report items, and complex analysis of these data as the field moves more in a PBT direction.

While PBT has the potential to reform the foundations of clinical practice, it is important to treat its value as a hypothesis that is as yet unproven. The change in direction it suggests is profound. Methods that have adopted a process-focused development strategy have been successful (Hayes et al., 2022), and some supportive early randomized trials of PBT methods have appeared (Ong et al., 2022), but that does not mean that adopting a PBT approach will necessarily lead to greater efficacy in psychological intervention writ large. Many case examples, clinical trials, and laboratory experiments with diverse populations will be needed to put empirical muscle on PBT’s theoretical skeleton. Thus, we offer the present case as a useful beginning example. We hope this paper will be the first of many to evaluate the efficacy and feasibility of a process-based approach. That is the only way to determine whether PBT can live up to its field-changing potential.

## Ethics statement

The studies involving human participants were reviewed and approved by the Boston University Charles River Campus Institutional Review Board. The patients/participants provided their written informed consent to participate in this study.

## Author contributions

CO analyzed the data. SCH and SGH provided clinical supervision. All authors contributed to case conceptualization and manuscript write-up.
