# Supplementary material for: A process-based approach to cognitive behavioral therapy: A theory-based case illustration
Source: Front Psychol. 2022 Oct 25;13:1002849. doi: 10.3389/fpsyg.2022.1002849 (PMC9642026; doi:10.3389/fpsyg.2022.1002849)
Supplement: Supplementary file 1 [file Data_Sheet_1.docx]

Supplementary Material

# Supplementary Tables

Table S1

Beta Estimates for Amy’s Network at the Start of Treatment

|  | Hypervigilance lag | Feeling at peace lag | Pressure to be responsible lag | Compulsion to problem solve lag | Hypervigilance | Feeling at peace | Pressure to be responsible | Compulsion to problem solve |
| --- | --- | --- | --- | --- | --- | --- | --- | --- |
| Hypervigilance | 0.2784 | 0 | 0 | 0 | 0 | 0 | 0 | 0.445 |
| Feeling at peace | 0 | -0.0185 | 0 | 0 | -0.7864 | 0 | 0 | 0 |
| Feeling at peace | 0 | 0 | 0.0096 | 0 | 0 | -0.7032 | 0 | 0 |
| Compulsion to problem solve | 0 | 0 | 0 | -0.0486 | 0 | 0 | 0.4957 | 0 |

Table S2

Beta Estimates for Amy’s Network at the End of Treatment

|  | Feeling empowered lag | Self-care lag | Flexible with standards lag | Aware of feelings lag | Connecting with others lag | Feeling empowered | Self-care | Flexible with standards | Aware of feelings | Connecting with others |
| --- | --- | --- | --- | --- | --- | --- | --- | --- | --- | --- |
| Feeling empowered | 0.5585 | 0 | 0 | 0 | 0 | 0 | 0 | 0 | 0 | 0.3165 |
| Self-care | 0 | 0.1566 | 0 | 0 | 0 | 0.2764 | 0 | 0.3349 | 0 | 0 |
| Flexible with standards | 0 | 0 | 0.4094 | 0 | 0 | 0.4303 | 0 | 0 | 0 | 0 |
| Aware of feelings | 0 | 0 | 0 | 0.3119 | 0 | 0 | 0.3424 | 0.1809 | 0 | 0 |
| Connecting with others | 0 | 0.236 | 0 | 0 | 0.2188 | 0 | 0 | 0.2506 | 0 | 0 |
